# Focal Ischemic Injury with Complex Middle Cerebral Artery in Stroke-Prone Spontaneously Hypertensive Rats with Loss-Of-Function in NADPH Oxidases

**DOI:** 10.1371/journal.pone.0138551

**Published:** 2015-09-21

**Authors:** Hiroshi Yao, Mohammed Zubaerul Ferdaus, Hasan Md. Zahid, Hiroki Ohara, Tatsuo Nakahara, Toru Nabika

**Affiliations:** 1 Laboratory for Neurochemistry, National Hospital Organization Hizen Psychiatric Center, Saga, Japan; 2 Department of Functional Pathology, Shimane University School of Medicine, Izumo, Japan; National Cerebral and Cardiovascular Center, JAPAN

## Abstract

By means of introgressing a loss-of-function mutation in the *p22phox* gene from the Matsumoto Eosinophilia Shinshu (MES) rat to stroke-prone spontaneously hypertensive rats (SHRSP), we constructed the SHRSP-based congenic strain lacking the P22PHOX expression (i.e., lacking NADPH oxidases [NOX] activities) (SHRSP.MES-*Cyba* ^*mes*^/Izm; hereafter referred to as SP.MES). To examine the effects of Nox activities on the focal ischemic injury or stroke, we performed middle cerebral artery (MCA) occlusion in this new congenic strain; the distal MCA was occluded by 561-nm laser-driven photothrombosis. Resting mean arterial blood pressure was significantly lower in SP.MES when compared with the control PM0/SHRSP (150±11 mmHg vs. 166±11 mmHg). Cerebral blood flow decreased to 37±13% in SP.MES and 35±17% in PM0/SHRSP at 10 min after MCA occlusion (not significant). Infarct volume determined at 24 h after MCA occlusion in SP.MES was 89±39 mm^3^, which was not significantly different from 83±35 mm^3^ in PM0/SHRSP. The distal MCA pattern was more complex in SP.MES (median 3, IQR 3–5) than PM0/SHRSP (median 2, IQR 1–3) (p = 0.001). Because more complex distal MCA is known to produce larger infarction after distal MCA occlusion in SHR, we adjusted for the branching pattern in an ANCOVA. The adjusted mean of infarct volume was significantly smaller in SP.MES compared with that in PM0/SHRSP (67 [95% CI 46 to 87] mm^3^ vs. 100 [95% CI 82 to 118] mm^3^, p = 0.032). Elimination of the P22PHOX expression induced complex distal MCA, which would suggest the presence of ‘loss of complexity’ induced by enhanced oxidative stress in SHRSP; infarct size in SP.MES—when adjusted for distal MCA complexity—was significantly attenuated compared with that in PM0/SHRSP. Therefore, the present results suggest that Nox is harmful for ischemic brain tissue.

## Introduction

The nicotinamide adenine dinucleotide phosphate (NADPH) oxidases (Nox)—only enzymes in terms of its primary function of generating superoxide—are the predominant source of reactive oxygen species [1]. So far, the Nox family is known to be composed of 7 catalytic subunits termed Nox 1 to 5 and Duox 1 and 2. Their physiological function is extremely diverse including host defense and inflammation, cellular signaling, gene expression, cellular death, cellular senescence, cell growth, oxygen sensing, angiogenesis, and so forth [2]. Nox is also involved in physiological functions of vasculature and hypertension [2,3], the latter of which is one of the major risk factors for stroke; superoxide rapidly interacts with nitric oxide to form peroxynitrite, resulting in vasoconstriction and increased blood pressure [3]. Basal and stimulated generation of superoxide from NADPH oxidases is 1 to 2 orders of magnitude higher in intracranial cerebral arteries than in systemic arteries, and activation of Nox in cerebral arteries can cause vasodilation under normal conditions [4]. Focal ischemia based on the middle cerebral artery (MCA) occlusion is the model of human acute brain infarction caused by occlusion of major cerebral arteries [5]. Because no specific Nox inhibitors exist at this moment, studies using Nox-knockout mice are dominant as the approach for assessing the effects of Nox on stroke pathophysiology. Nox-knockout mice generally showed protection against focal ischemia or experimental stroke (i.e., the evidence for detrimental effects of Nox on stroke outcome) [1,6].

It is not clear, however, whether Nox affects the stroke outcome directly through increasing the oxidative stress at the site of ischemia, or through modifying physiological variables such as blood pressure and cerebral blood flow (CBF) [5,7]. Because of a technical problem in measurement of physiological variables in mice due to their small body size, it is often difficult to address this issue in mice. Furthermore, there are methodological difficulties in determining CBF with laser-Doppler flowmetry in mice, because it is difficult to place laser-Doppler probe avoiding superficial veins and arterioles due to a small size of the brain and thus the high density of blood vessels. Therefore, we attempted to construct a new rat model lacking Nox activities. The Matsumoto Eosinophilia Shinshu (MES) rat was found to harbor a deletion of four nucleotides in the intron 4 of the cytochrome b(-245), alpha polypeptide (*Cyba*) gene, which resulted in an abnormal splicing. As *Cyba* encodes *p22phox*, this strain lacked expression of the P22PHOX protein and showed loss of Nox activities [8]. By introgressing the mutated *Cyba* gene from the MES strain, we constructed a congenic strain lacking the Nox activities on the background of the stroke-prone spontaneously hypertensive rat (SHRSP). We hypothesized that the congenic removal of P22PHOX and subsequent loss-of-function in Nox would attenuate infarct size produced by distal MCA occlusion. In this communication, we performed MCA occlusion in this new congenic strain, and examined the effects of deprivation of Nox activities on the focal ischemic injury.

## Materials and Methods

The National Hospital Organization Hizen Psychiatric Center Institutional Review Board (the Animal Care and Use Committee) approved all animal experimental and maintenance procedures (approval number: 26–6).

### Construction of congenic strains

**Fig 1:**
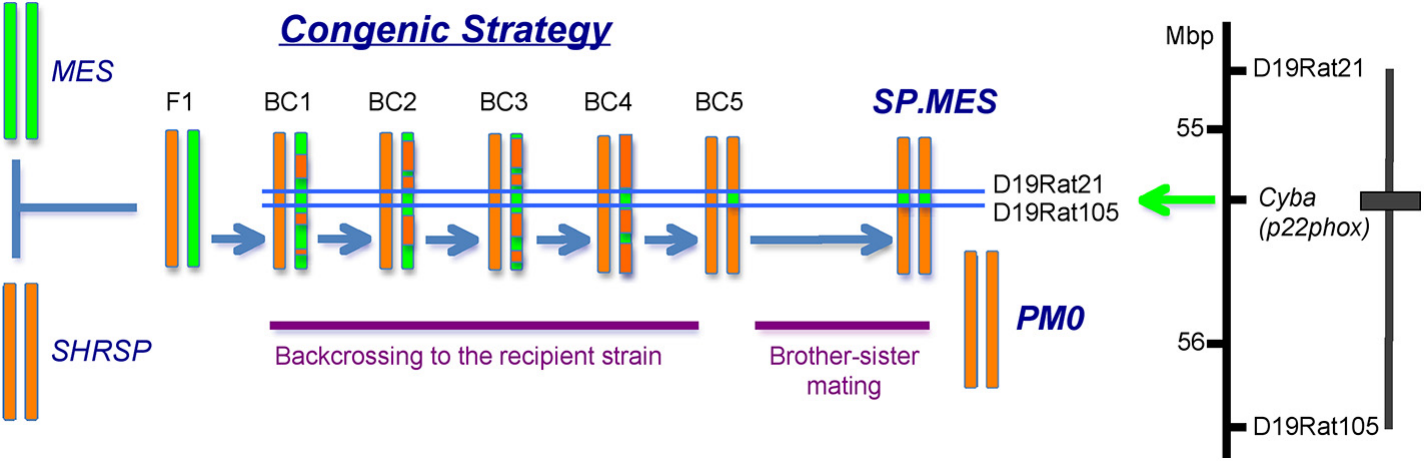
Construction of congenic strains. The mutated *Cyba* allele of MES was introgressed onto the genomic background of SHRSP. Using the MES strain as the donor and SHRSP/Izm as the recipient, we constructed a congenic strain without p22phox protein with SHRSP/Izm background by the speed congenic strategy. The target region was between D19Rat21 and D19Rat105. After 5 generations of backcrossing, all the 140 background simple sequence repeat markers were confirmed to be homozygous for the recipient allele, and then the congenic strain with the target region homozygous for the donor strain (i.e., MES) was obtained through brother-sister matings (SP.MES). Rats with the target region homozygous for the recipient strain (i.e., SHRSP/Izm) were used as control (PM0). The congenic region was maximally 1.7-Mbp between the two markers. The box indicates the region from the MES rat, and the vertical bar shows the region containing the recombination break point.

A congenic SHRSP harboring the mutated *Cyba* of the MES rat (SHRSP.MES-*Cyba* ^*mes*^/Izm; referred to as SP.MES hereafter) was constructed by the speed congenic strategy [9]. Genotyping of the *Cyba* allele was performed by PCR as described in the previous report [8]. One hundred and forty markers throughout the genome were examined to check the genotype of the background genome of the rats. Consequently, SP.MES harboring a 1.7-Mbp genomic fragment of MES including the *Cyba* locus on the SHRSP background was established (***Fig 1***) [7,9]. At the final step of the construction of the congenic strain, rats homozygous for the wild type allele of *Cyba* were simultaneously obtained as well, which were used as a control for the SP.MES (hereafter referred to as PM0).

As reported in the original paper [8], the 55-bp insertion in the mRNA of p22phox was confirmed by RT-PCR (S1 Fig); loss of P22PHOX expression was confirmed by Western blotting in SP.MES (S2 Fig).

PM0 (n = 9) and SHRSP (n = 5) were combined as one group for statistical analysis because they shared an identical genetic background, and there were no differences in body weight (285±25 g and 269±25 g, respectively) and resting MABP (165±14 mmHg and 165±8 mmHg, respectively) between the two strains.

### Photothrombotic distal MCA occlusion

**Fig 2:**
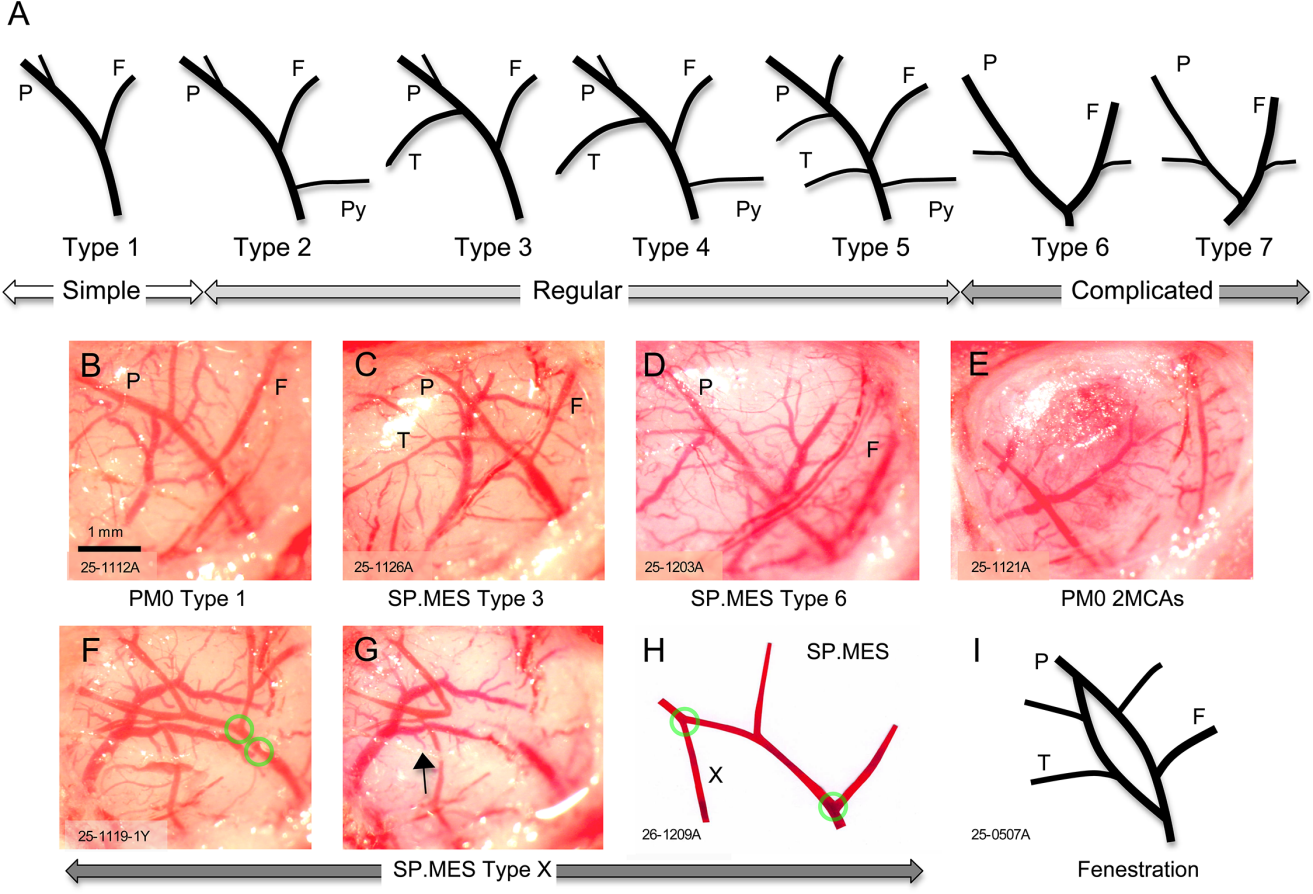
Branching pattern of diatal MCA. (A) The branching pattern of distal middle cerebral artery (MCA) modified from Cai H et al. (*Stroke* 1998;29:1982–1987). (B)-(E) Examples of distal middle cerebral artery (MCA) pattern (B, Type 1; C, Type 3; D, Type 6; E, 2MCAs) are presented. (F)-(H) In 2 SP.MES rats, we found extremely atypical distal MCA (named as Type X). When the distal MCA was occluded in a routine manner (F), blood flow was maintained through an aberrant vessel (arrow). In the second case, however, we could block this aberrant blood flow (X) by placing the laser beam at 2 separate points (H). (I) Fenestration of distal MCA in a SP.MES rat. We excluded atypical distal MCAs (E-I, i.e., 2MCAs, Type X, and fenestration) from the analysis.

A total of 31 male rats (11–20 weeks old) were used in this study. The branching pattern of distal MCA was determined according to the criterion as described previously [10] with slight modification (***Fig 2A***).

Rats were anesthetized with halothane (4% for induction, 1.5% during the surgical preparation with a face mask, 0.75% after intubation, and 0.5% for maintenance) in a mixture of 70% nitrous oxide and 30% oxygen. By means of photothrombosis, distal MCA was occluded as previously described.^12^ Briefly, a diode laser operating at 561 nm (Sapphire 561–50 CW CDRH; COHERENT JAPAN, INC., Tokyo, Japan) was used to irradiate the distal MCA. The laser beam was focused with a 30-cm-f.l. convex lens (KPX 112; Newport Corporation, Irvine, CA, USA) and positioned onto the distal MCA after reflection from a mirror. The photosensitizing dye rose bengal (15 mg/mL in 0.9% saline; Wako Pure Chemical Industries Ltd, Osaka, Japan) was administered intravenously to a body dose of 20 mg/kg over 90 sec starting simultaneously with laser irradiation at a power of 20 mW.

Regional CBF was assessed by means of laser-Doppler flowmetry (ALF21D, Advance Co. Ltd, Tokyo, Japan). The laser-Doppler probe was positioned over the penumbra area supplied by the MCA (at 2 mm posterior and 3 mm lateral to the bregma above the intact dura) as previously described [5,11].

The rats were monitored during the 1-h survival period (until approximately 2 h after distal MCA occlusion) and every 3 hours in the next day. Twenty-four h after distal MCA occlusion, the rat was decapitated under deep anesthesia. The brain was rapidly removed, cooled in ice-cold saline for 3 min, and was cut into 2 mm-thick coronal sections in a cutting block. The slices were stained with 1% 2,3,5-triphenyltetrazolium chloride (Wako Pure Chemical Industries Ltd., Osaka, Japan) at 37°C for 30 min in the dark. The infarct area was measured in each slice with NIH Image software (ImageJ 1.48v).

We performed a retrospective analysis of distal MCA pattern and infarct volume after distal MCA occlusion done in our laboratory during 2005–2012.

### Statistical analysis

Data are expressed as mean±S.D. We used the unpaired two-tailed t-test or Mann-Whitney u-test as appropriate to perform univariate comparisons of groups. Resting and Intra-ischemic MABP and CBF values were compared with use of 2-way analysis of variance (ANOVA) followed by Bonferroni post hoc test. Independent dose-effect relationship of infarct volume between the groups was investigated by means of an analysis of covariance (ANCOVA) (a general linear model) with distal MCA pattern as covariate; including independent variable/covariate interactions in an initial analysis step tested the ANCOVA homogeneity of regression assumption. All analyses were carried out with IBM SPSS 18.0.

## Results

**Fig 3:**
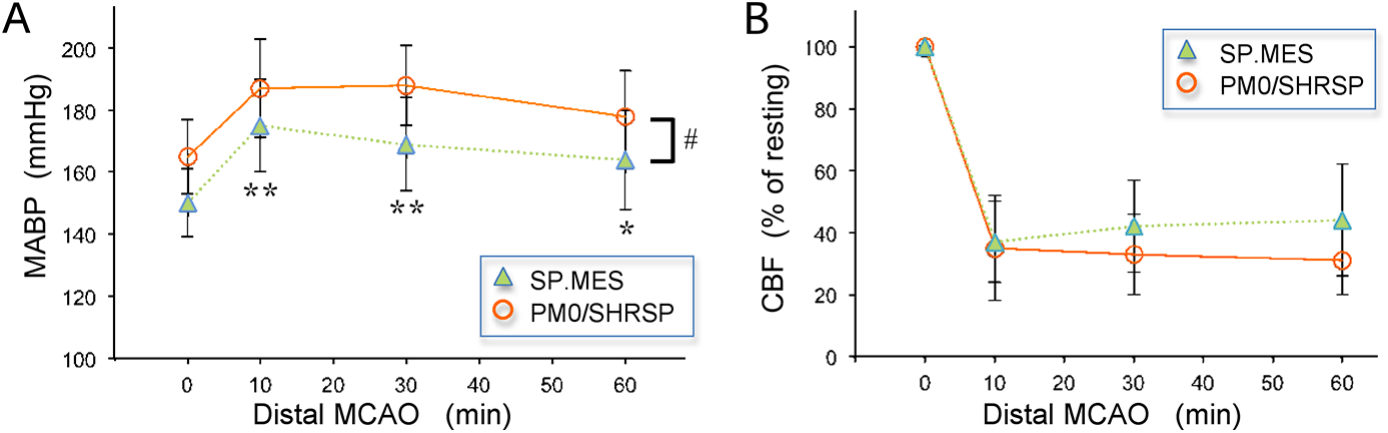
Mean arterial blood pressure (MABP) and Cerebral blood flow (CBF). (A) Changes in MABP before and after distal MCA occlusion: 2-way ANOVA revealed a group difference and an effect of time (**p<0.001 and *p<0.05 vs. 0 min, #p<0.001 between the groups, Values are mean±S.D.). (B) 2-way ANOVA did not show a significant group difference in CBF after MCA occlusion. Data are expressed as mean±S.D.

**Fig 4:**
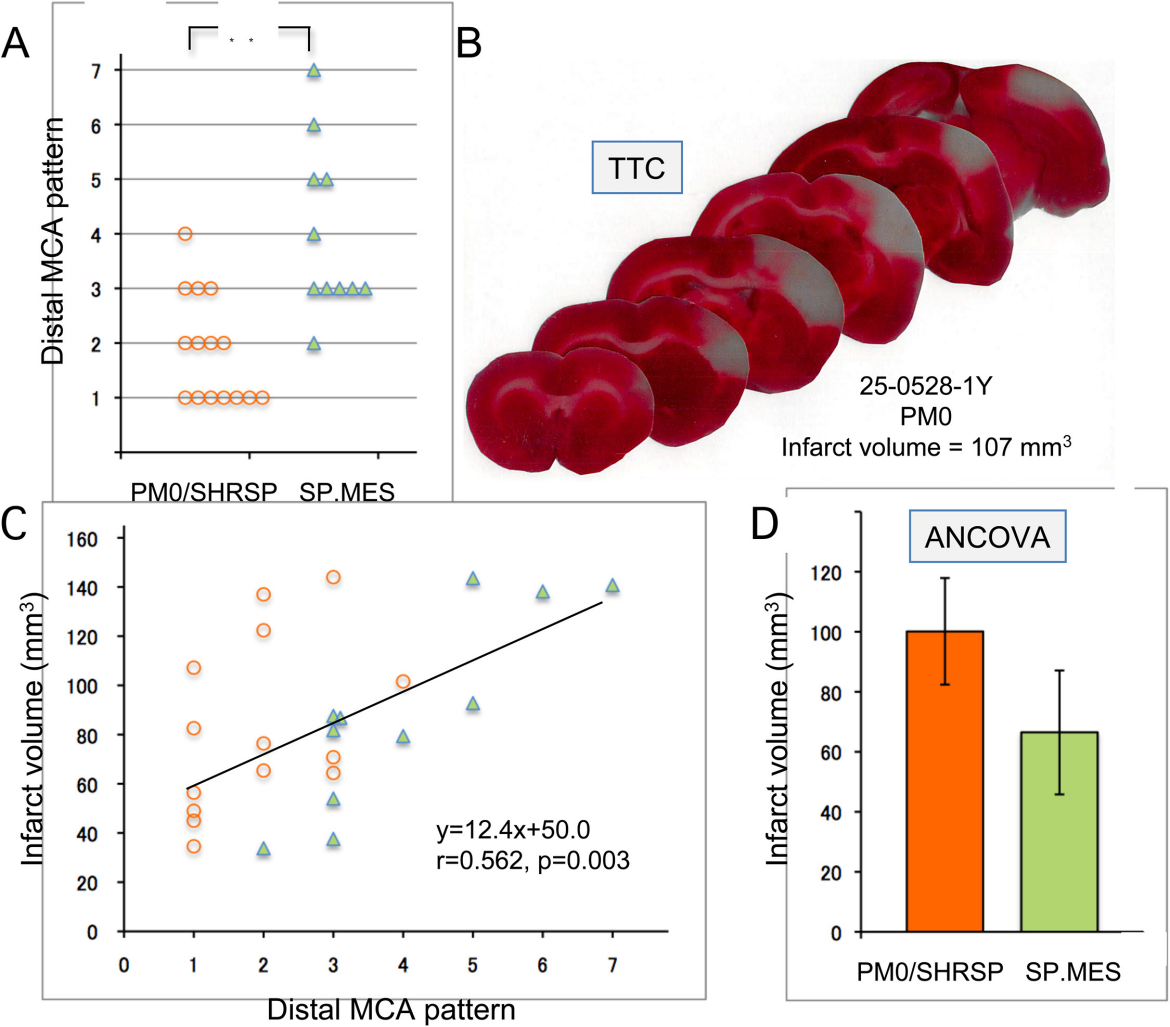
Distal middle cerebral artery (MCA) pattern and infarct volume. (A) Distal MCA patterns were more complex in SP.MES (median 3, interquartile range [IQR] 3–5) than PM0/SHRSP (median 2, IQR 1–3) (Mann-Whitney u-test, p = 0.001). (B) Representative brain sections stained with 2,3,5-triphenyltetrazolium chloride (TTC) from PM0 subjected to distal middle cerebral artery occlusion (MCAO) 24 h earlier. (C) Infarct volume in the SP.MES group was 89±39 mm^3^, which was not significantly different from 83±35 mm^3^ in the PM0/SHRSP group. Infarct volume was linearly correlated with distal MCA branching pattern. (D) The adjusted mean of infarct volume was significantly smaller in SP.MES compared with that in PM0/SHRSP (67 [95% CI 46 to 87] mm^3^ vs. 100 [95% CI 82 to 118] mm^3^, p = 0.032). Data are expressed as mean±S.D.

Body weight was significantly lower in SP.MES than PM0/SHRSP (240±27 g vs. 276±27 g, p = 0.002). Baseline physiological variables in SP.MES and PM0/SHRSP are presented in ***Table 1***. Resting MABP in SP.MES was slightly but significantly lower than that in PM0/SHRSP (p = 0.006). After MCA occlusion, blood pressure increased in both groups, but the intra-ischemic MABP levels were still lower in SP.MES than in PM0/SHRSP (***Fig 3A***, 2-way ANOVA, p<0.001 for a group difference and an effect of time). CBF decreased to 37±13% in SP.MES and 35±17% in PM0/SHRSP at 10 min after MCA occlusion (***Fig 3B***, 2-way ANOVA, not significant). The changes in CBF from 10 min to 60 min after MCA occlusion were 7±8% and -4±8% in SP.MES and PM0/SHRSP, respectively (p = 0.006). One of 10 PM0 rats died during the 24 h of survival period after MCA occlusion. Infarct volume in the SP.MES group was 89±39 mm^3^, which was not significantly different from 83±35 mm^3^ in the PM0/SHRSP group. The infarct volume was linearly correlated with the distal MCA branching pattern (Fig 4C). Variability of infarct volume was relatively large in the present study compared with our previous experiments with SHRSP of 5 months old, probably because the age of rats in the present study was relatively young (11 to 20 weeks), during which time a substantial increase in infarct volume and thus variability was observed [7]. The groups were, however, well matched for age in this study, hence age was not expected to affect the inter-strain difference of the infarct volume.

**Table 1:** Physiological variables in SP.MES and PM0/SHRSP.

		SP.MES	PM0/SHRSP
		n = 11	n = 14
Mean arterial blood pressure	(mmHg)	150±11*	165±13
Head temperature	(°C)	36.5±0.2	36.5±0.3
Rectal temperatue	(°C)	37.3±0.2	37.4±0.3
Blood gases			
pCO_2_	(mmHg)	36.7±1.9	38.8±3.3
pO_2_	(mmHg)	115±11	114±14
pH		7.42±0.05	7.44±0.06
Fasting blood glucose	(mmol/L)	6.27±1.05	6.38±0.72
Electrolytes			
Sodium	(mmol/L)	140±2	140±4
Potassium	(mmol/L)	3.8±0.2	3.6±0.2

Values are mean±S.D.

*p = 0.006 vs. PM0/SHRSP, unpaired t-test.

**Fig 5:**
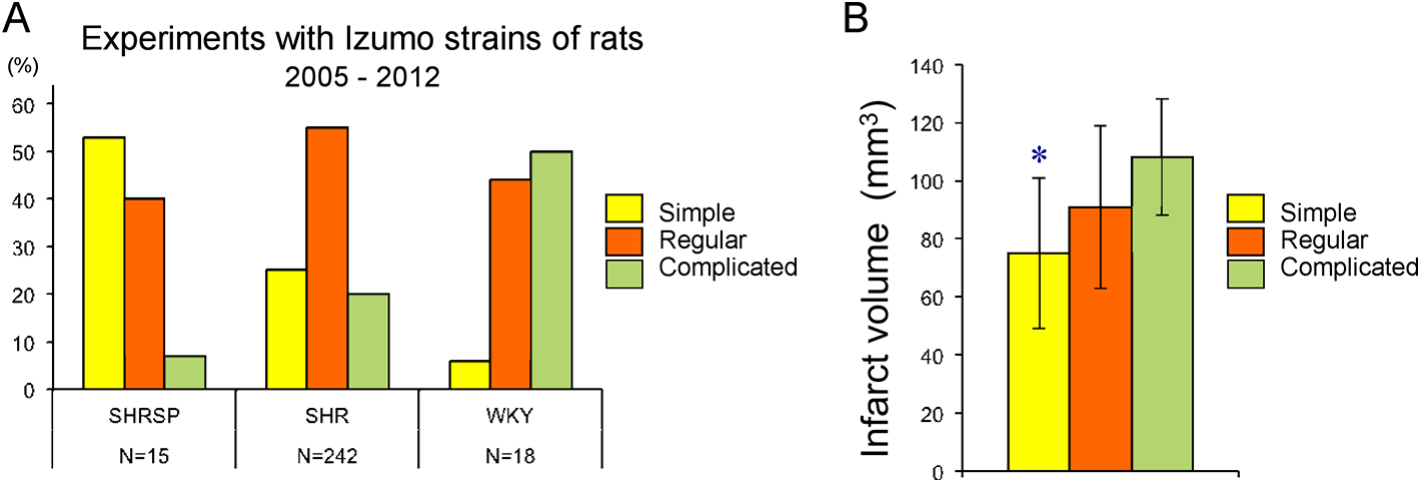
Retrospective analysis. (A) the frequency of distal MCA pattern in male spontaneously hypertensive rats (SHR),stroke-prone SHR (SHRSP), and Wistar-Kyoto rats (WKY). (B) infarct volume after distal MCA occlusion in SHR (5–7 months old, male) with simple (N = 16), regular (N = 25), or complicated (N = 8) MCA. *p = 0.017 vs. complicated, ANOVA & post-hoc Bonferroin test.

Examples of distal MCA are presented in ***Fig 2B–2G***: distal MCA is considered more complicated when the number of classification for the distal MCA pattern increases from Type I to Type VII (i.e., more branches and with tortuosity). Two PM0 rat with former Type VIII distal MCA (i.e., 2 MCAs), 2 SP.MES rats with Type X, and 1 SP.MES rat with fenestration of distal MCA were excluded from the analysis **(*Fig 2E–2I*)**. Distal MCA patterns were more complex in SP.MES (median 3, interquartile range [IQR] 3–5) than PM0/SHRSP (median 2, IQR 1–3) (***Fig 4A***, Mann-Whitney u-test, p = 0.001). In a retrospective analysis of distal MCA in the izumo (Izm) strains of rats, the simple pattern was more prevalent in SHRSP/Izm, while Wistar-Kyoto rats (WKY)/Izm had more complicated patterns of distal MCA (***Fig 5***). SHR/Izm showed an intermediate feature between SHRSP/Izm and WKY/Izm. Infarct volume following MCA occlusion in SHR/Izm with simple distal MCA was 75±26 mm^3^, which was significantly smaller than 108±20 mm^3^ in SHR/Izm with complicated distal MCA (p = 0.017, ANOVA and Bonferroni post hoc test) (***Fig 5***). Physiological variables were not significantly different among the groups (***Table 2***). Therefore, complicated distal MCA is a predictor for large infarction after distal MCA occlusion. As expected from this retrospective analysis and our previous experience^11^—when adjusted for the branching pattern in an ANCOVA—the adjusted mean of infarct volume was significantly smaller in SP.MES compared with that in PM0/SHRSP (67 [95% CI 46 to 87] mm^3^ vs. 100 [95% CI 82 to 118] mm^3^, p = 0.032) **(*Fig 4C*)**.

**Table 2:** Physiological variables in SHR/Izm (5–7 months old).

		Simple	Regular	Complicated
		n = 16	n = 25	n = 8
Mean arterial blood pressure	(mmHg)	173±15	166±9	167±9
Head temperature	(°C)	36.5±0.2	36.4±0.3	36.4±0.2
Rectal temperatue	(°C)	37.1±0.4	37.1±0.4	37.3±0.4
Blood gases				
pCO_2_	(mmHg)	38.8±3.5	38.7±2.9	38.4±2.5
pO_2_	(mmHg)	109±9	108±14	113±12
pH		7.41±0.03	7.42±0.02	7.41±0.02
Fasting blood glucose	(mmol/L)	7.73±1.08	7.14±1.10	7.60±1.01

Values are mean±S.D.

## Discussion

In the present study, we unexpectedly found that presumed functional loss of Nox associated with the absence of P22PHOX protein did not mitigate the size of infarction produced by distal MCA occlusion. Superoxide overproduction during cerebral ischemia deteriorates stroke outcome [1,6]. We observed a slight but significant reduction in blood pressure in SP.MES; chronic reductions of blood pressure by anti-hypertensive treatments or congenic removal of a quantitative trait locus for blood pressure were previously reported to attenuate infarction produced by MCA occlusion in SHRSP [7,12]. We therefore expected that decreased superoxide production and lower resting blood pressure would decrease the infarct size in SP.MES. However, more complex MCA, observed in SP.MES, might have resulted in larger infarction, since an ANCOVA showed a substantial reduction in infarct volume in SP.MES when adjusted for the distal MCA pattern or complexity. These divergent effects of phenotypes caused by Nox dysfunction may be the reason for the negative result in terms of similar infarct size between SP.MES and PM0/SHRSP.

The vascular system is probably the most prominent instance of hierarchical tubular network characterized by a topological feature (i.e., branching) [13]. Removal of the P22PHOX expression in SHRSP made the configuration of MCA more complex in SP.MES compared with PM0/SHRSP. To our knowledge, a direct involvement of reactive oxygen species or of the Nox activity has not been suggested in the context of branching morphogenesis of blood vessels. Lipsitz and Goldberger proposed the theory of age-related loss of complexity, in which the fractal-like (self-similar) branching architecture was implied as a possible subject for the nonlinear analyses [14]. Interestingly, decreased branching complexity of retinal vessels was associated with increasing age and lacunar infarction in stroke patients, which suggested that more pathological status brought less complex blood vessels or loss of complexity [15]. SHRSP dominantly showed ‘loss of complexity’ of MCA compared with its normotensive counterpart (WKY) (***Fig 5A***). Since it is widely believed that SHRSP had a higher level of the oxidative stress [16], the hypothesis seems intuitively attractive that increased levels of oxidative stress in SHRSP induced the simple pattern of MCA.

In the present study, we showed that beneficial effects of *p22phox* ‘knockout’ on the infarct size induced by MCA occlusion were negligible as a total. This might be because two events with opposing effects (i.e., complex distal MCA and reduced blood pressure) might have counteracted each other. The complex pattern of MCA observed in SP.MES may cause larger infarction as shown in our previous study [10]; we confirmed this phenomenon in our retrospective analysis of experiments performed between 2005 and 2012 (***Fig 5***). On the other hand, reduced blood pressure in SP.MES would predict smaller infarction as discussed above [5,7,12]. The former might counteract the latter in SP.MES, cancelling the beneficial effects of lowering blood pressure and/or the presumed reduction in oxidative stress against focal ischemic injury. Another interpretation would be that, as P22PHOX is an essential subunit for the activity of multiple members of the Nox family [2], several subtypes of Nox might play opposite roles in the ischemic brain injury. Previous studies on knockout mice suggested different roles of the Nox subtypes on ischemic brain damage; Nox2 seemed to have deleterious effects [17–21], whereas effects of Nox1 and 4 were controversial [22–25]. Finally, the experimental condition of permanent MCA occlusion might be insufficient to activate Nox as to the level of exacerbating the brain ischemic injury; the infarct volume was not dependent on the Nox2 activity in mice when reperfusion was not achieved after MCA occlusion [26].

Our present study showed that CBF decreased to a similar level immediately after distal MCA occlusion in PM0/SHRSP and SP.MES groups, and then increased slightly only in the SP.MES group, which suggested improved vasodilator function caused by the loss of Nox activity. However, CBF after suture MCA occlusion was not different between Nox-deficient and wild type mice in all the studies with CBF measurements [18–20,22,26]. In general, CBF reduction after suture occlusion was severe (approximately 20% or less of baseline), which indicated that CBF was determined at ischemic core rather than collaterally perfused zone or penumbra around the ischemic core. Another possibility would be that knocking out one subtype of Nox families may not be sufficient to alleviate vascular function under the conditions of suture occlusion in the previous studies [18–20,22,26]. By contrast, multiple Nox members were assumed to loose their function due to lack of P22PHOX in SP.MES in the present study.

This study has several limitations. First, the congenic region was estimated as 1.4-Mbp in maximum, which harbored tens of genes. Although the congenic strategy used in this study successfully removed the P22PHOX expression from SHRSP, we cannot exclude the possibility that abnormalities in other genes in the 1.4-Mbp region might have influenced the results—the attenuated hypertension and the more complex distal MCA—observed in the present study. Second, we used a permanent MCA occlusion in the present study and hence reperfusion injury was not considered. The effects of *p22phox* ‘knock-out’ might be much evident in an ischemia-reperfusion model. Third, we could not address which subtype of Nox played a major role in the ischemic brain injury based on the results of the present study. Recently, genome-editing technologies such as Talen and CRISPR/CAS9, were successfully applied to rats [27,28]. It is attractive to construct ‘knockout rats’ for each member of the Nox family to evaluate roles of individual Nox subtype in ischemic brain injury as well as branching patterns of MCA.

In conclusion, the congenic exchange of the region including the p22phox gene of SHRSP with that of SP.MES induced complex distal MCA; enhanced oxidative stress in SHRSP might have induced ‘loss of complexity’ or simple distal MCA. Infarct size in SP.MES—when adjusted for distal MCA complexity—was significantly attenuated compared with that in PM0/SHRSP. Therefore, as most experimental studies demonstrated, Nox was considered to be harmful for ischemic brain tissue based on the present results. The mechanisms of Nox-related ischemic injury, however, are not clear from the present study. In a future study, the effect of P22PHOX deprivation on infarction under transient focal ischemia (i.e., reperfusion injury) should be addressed.

## Supporting Information

S1 Fig RT-PCR for p22phox. The mutated allele of *P22phox* in SP.MES; RT-PCR was performed on mRNA extracted from the hindbrain tissue. The primers used in the PCR were 5′-TTGTTGCAGGTGTGCTCATC-3′ (forward) and 5′-GTTTAGGCTCAATGGGAGTCC-3′ (reverse). As shown in the panel, SP.MES had a longer PCR product indicating the 55-bps insertion in the *P22phox* gene.
(TIF)

S2 Fig Western blot for P22PHOX. The Western blot of P22PHOX—the protein sample was prepared from the spleen—clearly indicates no protein expression in SP.MES. The western blotting was performed with a rabbit anti-P22PHOX antibody (Santa Cruz Biotechnology, Santa Cruz, CA, USA) and anti-b-actin (clone AC-15, Sigma-Aldrich, St. Louis, MO, USA).
(TIF)
