# Supplementary figures and images for: Focal Ischemic Injury with Complex Middle Cerebral Artery in Stroke-Prone Spontaneously Hypertensive Rats with Loss-Of-Function in NADPH Oxidases
Source: PLoS One. 2015 Sep 21;10(9):e0138551. doi: 10.1371/journal.pone.0138551 (PMC4577106; doi:10.1371/journal.pone.0138551)

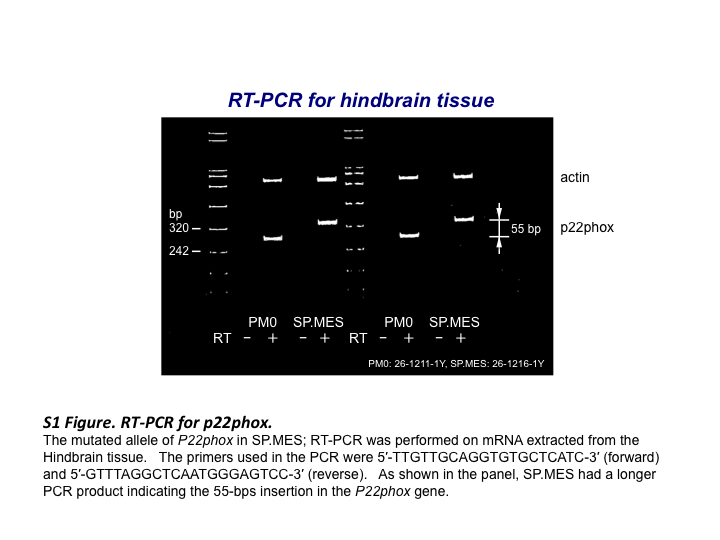

Supplement: S1 Fig — The mutated allele of P22phox in SP.MES; RT-PCR was performed on mRNA extracted from the hindbrain tissue. The primers used in the PCR were 5′-TTGTTGCAGGTGTGCTCATC-3′ (forward) and 5′-GTTTAGGCTCAATGGGAGTCC-3′ (reverse). As shown in the panel, SP.MES had a longer PCR product indicating the 55-bps insertion in the P22phox gene. (TIF) [file pone.0138551.s001.tif]

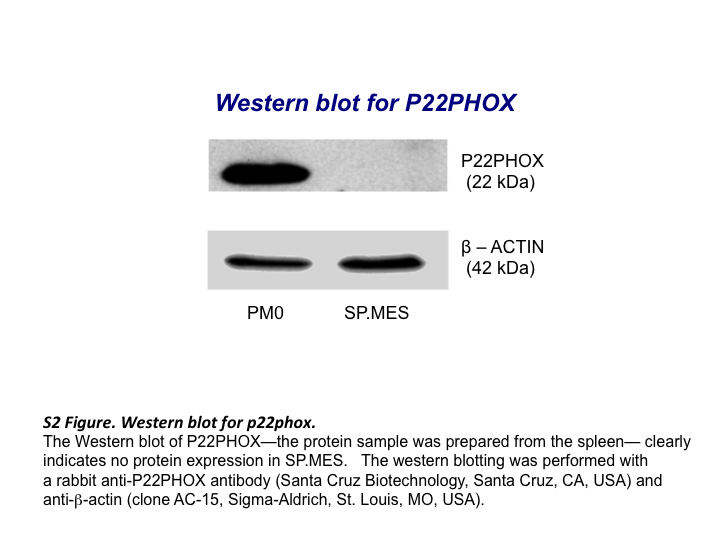

Supplement: S2 Fig — The Western blot of P22PHOX—the protein sample was prepared from the spleen—clearly indicates no protein expression in SP.MES. The western blotting was performed with a rabbit anti-P22PHOX antibody (Santa Cruz Biotechnology, Santa Cruz, CA, USA) and anti-b-actin (clone AC-15, Sigma-Aldrich, St. Louis, MO, USA). (TIF) [file pone.0138551.s002.tif]
